# Hands washing, glove use, and avoiding recontamination before aseptic procedures at birth: A multicenter time-and-motion study conducted in Zanzibar

**DOI:** 10.1016/j.ajic.2018.07.021

**Published:** 2019-02

**Authors:** Giorgia Gon, Marijn de Bruin, Mícheál de Barra, Said M. Ali, Oona M. Campbell, Wendy J. Graham, Mohammed Juma, Stephen Nash, Claire Kilpatrick, Loveday Penn-Kekana, Sandra Virgo, Susannah Woodd

**Affiliations:** a London School of Hygiene and Tropical Medicine, Faculty of Epidemiology and Population Health, London, United Kingdom; b University of Aberdeen, Institute of Applied Health Sciences, Aberdeen, United Kingdom; c Brunel University London, Department of Life Sciences, Uxbridge, United Kingdom; d Public Health Laboratory-Ivo de Carneri, Chake Chake, Pemba, Zanzibar, Tanzania; e Consultant, World Health Organization IPC Global Unit, Service Delivery and Safety Department, Geneva, Switzerland

**Keywords:** Maternal health, Newborn health, Behavioral medicine, Labor ward, Tanzania, Hand hygiene

## Abstract

**Background:**

Our primary objective was to assess hand hygiene (HH) compliance before aseptic procedures among birth attendants in the 10 highest-volume facilities in Zanzibar. We also examined the extent to which recontamination contributes to poor HH. Recording exact recontamination occurrences is not possible using the existing World Health Organization HH audit tool.

**Methods:**

In this time-and-motion study, 3 trained coders used WOMBATv2 software to record the hand actions of all birth attendants present in the study sites. The percentage compliance and 95% confidence intervals (CIs) for individual behaviors (hand washing/rubbing, avoiding recontamination and glove use) and for behavioral sequences during labor and delivery were calculated.

**Results:**

We observed 104 birth attendants and 781 HH opportunities before aseptic procedures. Compliance with hand rubbing/washing was 24.6% (95% CI, 21.6-27.8). Only 9.6% (95% CI, 7.6-11.9) of birth attendants also donned gloves and avoided recontamination. Half of the time when rubbing/washing or glove donning was performed, hands were recontaminated prior to the aseptic procedure.

**Conclusions:**

In this study, HH compliance by birth attendants before aseptic procedures was poor. To our knowledge, this is the first study in a low- to middle-income country to show the large contribution to poor HH compliance from hand and glove recontamination before the procedure. Recontamination is an important driver of infection risk from poor HH. It should be understood for the purposes of improvement and therefore included in HH monitoring and interventions.

## Highlights

• To our knowledge, this is the first study to systematically examine recontamination after hand hygiene in a low- and middle-income country.
• Hand hygiene compliance before aseptic procedures was low (9.6%) among birth attendants in Zanzibar.
• Birth attendants did not avoid recontamination half of the time after hand rubbing/washing or glove donning.
• Recontamination should be investigated further to inform better behavior-change strategies.

Health care–associated infections (HAIs) in low- and middle-income countries (LMICs) affect an estimated 15% of patients, 3 times more than in Europe.^1^ For mothers and newborns in LMICs, where infection is already a leading cause of death,^2^, ^3^ the risk of HAIs could escalate with increasing health care facility newborn deliveries as well as substandard infection prevention standards.^4^

Hand hygiene (HH) is deemed the single most important behavior for preventing HAIs.^5^ Historical evidence suggests the importance of HH in reducing maternal infections in European hospitals, and recent studies support its value for newborns in LMICs.^6^ The World Health Organization (WHO) recommends Five Moments for Hand Hygiene (5MHH) during patient care.^7^ Among these, Moment 2—HH before clean/aseptic tasks when there is potential contact with patient's mucous membranes or nonintact skin—is considered the most significant for preventing bacterial transmission to patients, including the bloodstream, that could result in infection. During birth, this primarily occurs before and during a vaginal examination or delivery and related procedures.

Before these aseptic procedures, WHO guidelines require attendants to hand rub or wash, avoid recontaminating their hands, don gloves, and avoid recontaminating those gloves before starting the procedure.^7^ The current WHO HH Observation Form does not distinguish whether the failure to comply with the 5MHH stems from not hand rubbing/washing or from, for example, subsequently touching potentially unclean surfaces,^7^ thus negating the initial hand washing/rubbing action. Although successful multimodal interventions exist to improve HH, they require in-depth understanding of the context and achieve only variable long-term success.^5^^,^^7^, ^8^, ^9^ Determining whether birth attendants comply with any of the steps in the prescribed behavioral sequence and, more specifically, within the workflow in our context—Zanzibar, a region of Tanzania—is important to inform successful improvement interventions.

Therefore, our study aimed to examine the complex workflow in relation to hand hygiene and glove use undertaken by birth attendants in multiple high-volume labor wards in Zanzibar. Our specific research questions were:

1. What is the compliance with hand rubbing/washing (and then avoiding hand recontamination) and donning gloves (and then avoiding glove recontamination)?
2. Is variability of these behaviors primarily greater *between* birth attendants or *within* birth attendants across different HH opportunities?
3. To what extent does failure to avoid recontamination (as opposed to not hand rubbing/washing before a procedure) contribute to poor HH?
4. What behavior sequences do birth attendants undertake most often before aseptic procedures compared with the behavior sequence prescribed by WHO guidelines?

## METHODS

### Context

This study is part of the larger Hand-hygiene of Attendants for Newborn Deliveries and Survival (HANDS) project: a mixed-methods study investigating drivers of birth attendant HH. HANDS ran between November 2015 and April 2017 in the 10 highest-volume labor wards in Zanzibar, with average monthly delivery volumes ranging from 75 to 930 (Appendix A, available from https://doi.org/10.17037/DATA.00000778). The project was a partnership of the London School of Hygiene and Tropical Medicine, the University of Aberdeen, and the Public Health Laboratory of Pemba. Previous work in 8 of these maternity wards found that most had policies and basic infrastructure to perform HH, but only 50% received HH training in the previous year.^10^

### Study design and data collection

Within HANDS, we conducted a time-and-motion study wherein 3 observers recorded the hand actions (eg, procedures and hand touches on surfaces) of birth attendants 24 hours per day (1 data collector per 8-hour shift–morning, evening, and night), for a mode of 6 days (range, 5-14 days) per labor ward. Results are reported using the Strengthening the Reporting of Observational Studies in Epidemiology guidelines.^11^ All observers were trained midwives. Birth attendants were all staff involved in assisting deliveries, irrespective of cadre, including midwives and orderlies. Details of the tool, training, and data collection protocols can be requested from the authors.

To estimate an HH compliance of 10% with an absolute precision of ±3%, 768 HH opportunities were required. For the sample size calculation, we used the formula for estimating a proportion from a cross-sectional survey, with α = 0.05 and a design effect of 2, based on a survey in Benin of facility quality indicators.^12^ Using the reported number of deliveries in the 10 study facilities overall, we calculated the length of observation required to achieve this sample size.

Data were collected via tablets, precoded using WOMBATv2 software (© Centre for Health Systems and Safety Research, Macquarie University, Sydney, New South Wales).^13^, ^14^ An observation session began when an attendant started assisting a woman in labor. All observed hand actions were recorded as they occurred, and the time of each was automatically logged. A set of mutually exclusive actions was precoded and used specifically in this study. One attendant was observed per observation session, but multiple patients or procedures could be included. Multiple observation sessions were usually captured in 1 shift. To minimize the Hawthorne effect, attendants in all facilities but the one where the pilot occurred were told that the observation was about overall quality of care, not specifically HH.^15^

We trained on and piloted the observation tool over 2 weeks, following WHO guidelines.^7^, ^16^ During the first month of data collection, we also assessed interobserver agreement between pairs of data collectors (on 49 or 50 behaviors for each pair) and calculated kappa statistics. We provided tailored feedback to the data collectors based on these results.

### Ethics

This project was approved by the Zanzibar Medical Research and Ethics Committee and the London School of Hygiene and Tropical Medicine Research Ethics Committee. Consent was obtained from women (patients) either in writing in the antenatal ward prior to observation or verbally in the labor ward, with written consent obtained before discharge. Women were informed that the person being observed was the birth attendant and that no information would be collected on them. Consent to observe the birth attendants was granted by the Ministry of Health Zanzibar and obtained verbally from the birth attendants when the data collectors first visited the facility. All observed health care worker information was anonymized.

## Definitions

### HH opportunity

HH compliance was calculated as the number of times HH was performed divided by the number of opportunities when HH ought to occur. The opportunities in this study were procedures at birth that ought to be aseptic (Table 1). We termed a “delivery flow” as any sequence of these procedures occurring one after the other without a break and considered as *1* opportunity for HH. We defined these opportunities using available guidelines,^16^, ^17^, ^18^ unstructured observations in 4 of the study wards, and expert consultation. This aimed to capture realistic workflows within our setting and accurately observe HH according to WHO recommendations.

**Table 1:** List of aseptic procedures during a delivery flow

Wiping the vagina
Vaginal examination
Artificial rupture of membranes
Episiotomy
Catching the baby (delivering the baby)
Cord cutting and clamping
Cord traction
Manual removal of placenta*
Postdelivery vaginal examination
Suturing of the perineum*
Wiping baby clean
Urinary catheter insertion or removal

⁎ We allowed manual removal of the placenta or suturing to be considered within the delivery flow when these occurred before or after a vaginal examination, during postdelivery examination, or during vaginal wiping, or when manual removal of the placenta occurred after cord traction.

During a delivery flow, a birth attendant was permitted to undertake hand actions within the *patient zone*, defined for this study as the woman's perineal area and thighs, any clean or sterile equipment being used, and the newborn as it was caught and wiped (Table 2). The patient zone included the patient and some surfaces and items that were temporarily and exclusively dedicated to her, limiting the risk of transmitting pathogenic organisms.^17^ We excluded the delivery bed and trolley from the patient zone because previous work in Zanzibar found that these surfaces were often contaminated with bacteria.^10^ A break in the delivery flow, indicating a new HH opportunity, arose if an activity occurred that was not exclusive to the patient zone (eg, inserting an intravenous line, touching the patient beyond the zone, or leaving the room).

**Table 2:** Types of hand actions that did *not* indicate a new opportunity for HH

Touching the patient's thighs or perineal area and the newborn after birth
Touching her own (the attendant's) body*
Touching a clean† delivery surface–cloth or macintosh
Touching equipment contaminated only with the woman's own body fluids during the procedure
Touching other sterile or clean material (eg, cotton swabs or drying material already available in the area for patient care)‡
Performing an injection (oxytocin) or supporting breastfeeding
Carrying the placenta to be disposed (ie, “dragging” the patient zone)
Removing or adding gloves or rinsing hands with water,§ per WHO recommendations

*HH*, hand hygiene; *WHO*, World Health Organization.

^⁎^ Unconscious touches (eg, touching briefly her own face) are allowed by WHO guidelines (7). During the training, we did not differentiate between this type of unconscious gesture and a longer behavior (eg, standing with hands on hips for minutes). This recommendation assumed overall cleanliness and health of the birth attendant. These “permitted touches” did not include the birth attendant's clothes or gown.

^†^ Usually, a delivery surface was a large rectangular sheet of cloth or plastic (also called a macintosh) brought by the woman from her own household. The surface was presumed to be clean, provided it was not contaminated (eg, with a woman's feces or after falling on the floor). When the observer could not see what happened to the sheet, it was presumed to be clean.

^‡^ If these items were collected outside the patient zone, they were also allowed as long as the birth attendant did not touch any other surface while collecting these items. Any other hand touch was recorded as a separate action and would indicate a new opportunity.

^§^ We allowed for the donning or removal of gloves and rinsing hands with water only during the delivery flow (after the first procedure) without indicating a new HH opportunity. This is because the WHO Guidelines for Pregnancy and Childbirth suggest that birth attendants should change their gloves before cord cutting and clamping, without needing HH, or that they should wash their gloved hands,^18^ although this is not a recommendation of the WHO HH guidelines.

### Hand rubbing/washing, glove use, and recontamination

Before a delivery flow, a birth attendant should perform 4 behaviors sequentially, defined in our study as follows^7^:

1. Rub hands with alcohol-based hand rub or wash hands with soap and water (soap use was presumed if the observer could not see the action).
2. Avoid hand recontamination after rubbing/washing until gloves are donned (or until the procedure if gloves are not worn).
3. Don at least 1 glove.
4. Avoid glove recontamination before starting the delivery flow.

We defined recontamination of hands or gloves as any touch on potentially contaminated surfaces within the workflow; this included touching an unclean delivery surface (eg, a sheet that was in contact with the floor or with the woman's feces), unclean hand-drying material (eg, reusable material), the woman and newborn outside the defined patient zone, the woman's bed, trolley, unclean objects used during HH (eg, the sink tap or the bin), and *other* unclean surfaces, unless classified as outside the workflow (a full list of activities outside the workflow is shown in Appendix B, available from https://doi.org/10.17037/DATA.00000778). These touches were distinguished from a deliberate new activity outside the workflow that would lead to a new HH opportunity as per the 5MHH (eg, leaving the room or measuring blood pressure after completion of the aseptic procedure; see Appendix B, available from https://doi.org/10.17037/DATA.00000778).

When none of the 4 behaviors was implemented, we described the suboptimal glove-related behaviors practiced instead.

### Data cleaning and analyses

One author cleaned and checked the data for consistency. When multiple actions were recorded simultaneously, we used the actions related to the hygiene behaviors and procedures of interest above other actions (eg, leaving the room), leading to some loss of information. When contradictory information was reported about the same action (eg, if observers recorded both that soap was used and that they did not see soap being used), we coded the data as *inconsistent information*. For software interruptions during data collection, we followed the WOMBAT guidelines to clean time data.^14^ We censored opportunities with insufficient information on hand rubbing/washing glove use, and recontamination because they occurred too close to the start of a time-and-motion observation session.

We estimated percentage compliance (behavior performed over number of opportunities) and 95% confidence intervals (CIs) for the entire recommended behavior sequence (Behaviors 1-4), for partial completion of the sequence, and for each of the 4 hygiene behaviors individually. Behaviors 2 and 4 (avoid hand and glove recontamination) were, respectively, contingent on hand rubbing/washing (Behavior 1) and donning gloves (Behavior 3) (see Appendix C for numerators and denominators for each combination, available from https://doi.org/10.17037/DATA.00000778).

We calculated frequency of adequate rubbing/washing technique (right palm over left dorsum with interlaced fingers and vice versa (16) and duration (≥10 seconds, following the Zanzibar infection prevention guidelines). We also described surfaces touched during hand/glove recontamination. Finally, we described within- and between-individual variation for the 4 behaviors using bar charts and intracluster correlation coefficients (ICCs), restricted to attendants with ≥5 opportunities. The ICC is a measure of the relatedness of data. It accounts for this relatedness by comparing the variance within clusters with the variance between clusters.^19^ The ICC was calculated on the log odds scale from univariate logistic regression models accounting for individual-level clustering at the birth attendant level. G.G. coded all outcomes, and S.W. checked the coding. Analyses were performed using STATA v14 software (StataCorp LLC, College Station, TX).

## Data sharing

Anonymized data at the opportunity level are available in Appendix F, from https://doi.org/10.17037/DATA.00000778.

## RESULTS

### Dataset

We observed a total of 7,893 hand actions (including procedures, touches, and HH). After cleaning, the final results present the actions of 104 birth attendants across 10 facilities, with 4-18 attendants per facility. These data were collected during 336 observation sessions ranging from 13 minutes to 6 hours, 45 minutes, with a median time of 1 hour, 41 minutes. Each attendant was observed 1-9 times (observation sessions). The kappa statistic calculated for pairs of data collectors was good for 2 of 3 pairs at 0.93 and 0.90, but it was below the optimal level of 0.85 for 1 of the pairs, at 0.73.^14^ Tailored feedback was provided to data collectors based on these results.

### HH opportunities

There were 914 HH opportunities, of which 127 (13.9%) were censored because they occurred too close to the start of the observation period. Six HH opportunities were dropped because they had inconsistent information on HH. Our final dataset contained 781 HH opportunities.

### Compliance levels

Birth attendants hand rubbed/washed in 24.6% (95% CI, 21.6-27.8; 192/781) of opportunities, and 6.3% (12/192) of these instances were hand rubbing. Compliance with hand rubbing/washing did not vary much by observer or by shift—the CIs overlapped (Appendix D, available from https://doi.org/10.17037/DATA.00000778). Hand rubbing/washing was performed with adequate technique 30.7% (59/192) of the time, and 14.6% (160/192) of the time lasted ≥10 seconds (Appendix E, available from https://doi.org/10.17037/DATA.00000778). Birth attendants avoided hand recontamination after rubbing/washing in 68.8% (95% CI, 61.7-75.2; 28/192) of opportunities.

In 63.0% (95% CI, 59.5-66.4; 492/781) of opportunities, attendants added at least 1 glove before the procedure (with or without prior hand washing/rubbing). Of these, 61.8% (95% CI, 57.3-66.1; 304/492) avoided glove recontamination. Overall, birth attendants risked recontaminating their hands or gloves in 45.3% (95% CI, 40.9-49.8; 227/501) of the opportunities when rubbing/washing or glove donning occurred.

Consider now the actions that led to failures in avoiding glove or hand recontamination (Table 3). On average, 1.3 unclean touches occurred after hand washing/rubbing (standard deviation [SD] = 0.7; range, 1-4), and the most commonly touched surfaces were the glove packs and unclean hand-drying material. On average, 1.5 unclean touches occurred after adding gloves (SD = 0.5; range, 1-7), and the most commonly touched surfaces were the patient outside the defined patient zone and unclean delivery surfaces.

**Table 3:** Surfaces touched risking recontamination after hand rubbing/washing or glove use

Type of surface touched	After hand rubbing/washing,	After adding gloves,
	% (n)	% (n)
	(N* = 78)	(N* = 275)
Gloves pack	47.4 (37)	0
Unclean material when drying hands	20.5 (16)	0
Other unclean touches	16.7 (13)	16.4 (45)
Patient touched in areas that are *not* within the defined zone (ie, the pelvis and thighs or the newborn)	9.0 (7)	56.0 (154)
Personal bag	5.1 (4)	2.2 (6)
Unclean delivery surface (cloth or macintosh)	1.3 (1)	20.0 (55)
Patient bed	0	5.1 (14)
Waste bin	0	0.4 (1)

⁎ Overall number of touches performed when birth attendants did not avoid hand or glove recontamination. These touches are spread across 60 opportunities when birth attendants did not avoid hand recontamination, whereas these touches are spread across 187 opportunities when birth attendants did not avoid glove recontamination.

### Between-person and within-person variability

**Fig. 1:**
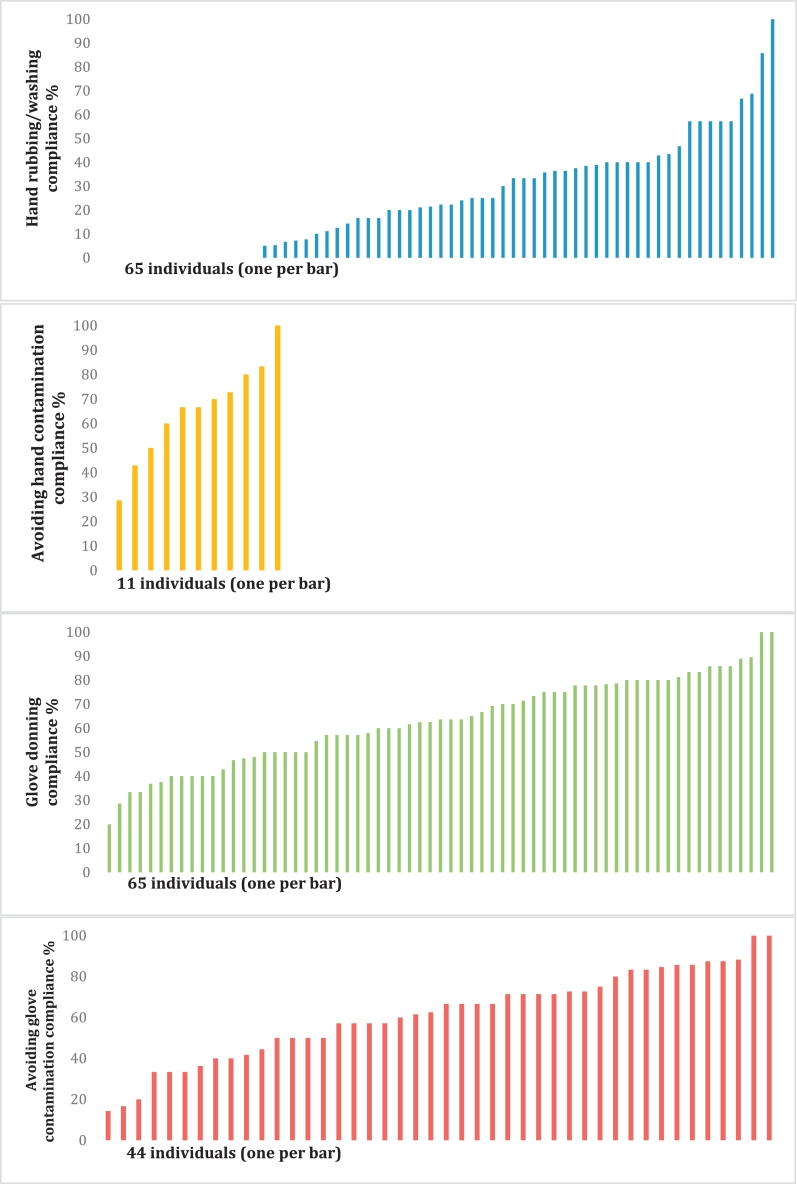
Distribution of individuals' compliance with hand rubbing/washing, glove use, and recontamination. NOTE. Only individuals with >5 opportunities were included in each of these graphs.

The 65 individuals with ≥5 HH opportunities contributed to the individual-level analyses of hand rubbing/washing (Behavior 1) and glove donning (Behavior 3) (Fig 1). However, recontamination could only be examined among 11 individuals who rubbed/washed and 44 individuals who donned gloves ≥5 times.

Fifteen attendants never rubbed/washed, 1 had 100% compliance, and the rest ranged between 5% and 85.7% compliance. The ICC indicates that most of the variation was within individuals (72%; 95% CI, 0.57-0.84) rather than between individuals (28%; 95% CI, 0.16-0.43). One attendant always avoided hand recontamination. The rest ranged between 28.6% and 83.3%. Most of the variation was within individuals rather than between individuals (10%; 95% CI, 0.01%-0.59%).

Two individuals never added new gloves before an aseptic procedure, and 5 individuals always did. The rest ranged between 10.5% and 88.2%. Almost all of the variation was within individuals (96%; 95% CI, 0.86-0.99) rather than between individuals (4%; 95% CI, 0.01-0.14). After glove donning, 2 individuals always avoided recontamination. The rest ranged between 14.3% and 88.2%. Only 8% (95% CI, 0.03-0.22) of the variation was between individuals, and most of the variation was within individuals (92%; 95% CI, 0.78-0.97). All ICC analyses were also carried out with all 104 individuals and yielded remarkably similar results.

### Behavior sequences

**Fig. 2:**
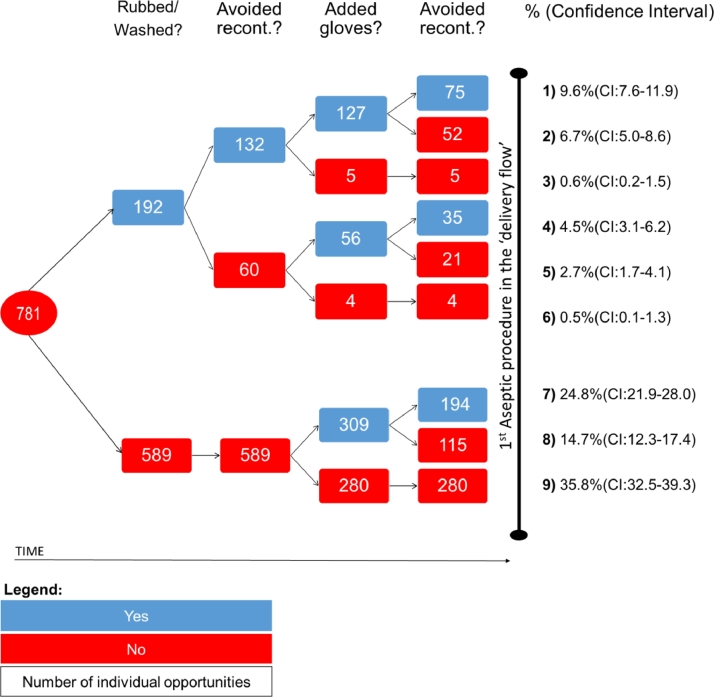
Behavior sequences for 781 hand hygiene opportunities. NOTE. This figure describes the 781 opportunities available in the dataset. For each opportunity, it outlines whether each of the 4 behaviors was performed. Percentages refer to the number of opportunities in the last column (eg, in the first sequence, 9.6% refers to 75/781). *Recont.,* recontamination.

Figure 2 presents the specific behavior sequences of birth attendants. Sequence 1, the WHO recommendation, was followed in only 9.6% (95% CI, 7.6-11.9) of opportunities. The most common practice, Sequence 9, was to perform none of the 4 behaviors (35.8%; 95% CI, 32.5-39.3), followed by donning gloves without hand rubbing/washing and avoiding glove recontamination (24.8%; 95% CI, 21.9-28.0) or not avoiding recontamination (14.7%; 95% CI, 12.3-17.4) (Appendix F, available from https://doi.org/10.17037/DATA.00000778).

In most opportunities in Sequence 9 (55.0%; 95% CI, 49.0-61.0; 154/280), attendants wore gloves used in a previous delivery flow. Other patterns are described in Appendix G, available from https://doi.org/10.17037/DATA.00000778.

## DISCUSSION

In this time-and-motion study of 104 birth attendants across the 10 highest-volume labor wards in Zanzibar, we observed 781 HH opportunities before aseptic procedures. Compliance with hand rubbing/washing occurred in a quarter of opportunities, but in only 9.6% of opportunities attendants also donned gloves and avoided hand and glove recontamination before the procedure, in accordance with WHO guidelines.^16^ Half the time, attendants either rubbed/washed hands or donned gloves that they subsequently touched unclean surfaces with, thus potentially recontaminating their hands and contributing substantially to poor HH compliance. The variation in behavior was much larger within individuals than between individuals, suggesting that these behaviors are not habitual.

Our findings of poor compliance are similar to those of other studies from LMICs. Low HH compliance (21%) before aseptic procedures was recently reported in a Nigerian hospital.^20^ In Indian labor wards, compliance before delivery was only 10.6%.^21^ A study from Iran reported similar levels during the second stage of labor.^22^ A study of a labor ward in Ghana reported that compliance ranged between 21% and 27% before aseptic procedures.^23^ In Zimbabwe, a study found that 62% of midwives never washed their hands before procedures.^24^ HH definitions vary in these studies, making direct comparison with our results challenging. However, all studies highlight extremely poor HH behavior.

Although for most opportunities birth attendants did not rub/wash hands, in two-thirds of opportunities they did wear at least 1 new glove for the procedure. In the remaining third, birth attendants adopted suboptimal glove-use behaviors that are not recommended^7^ but may imply an attempt at placing a barrier between the birth attendant's hands and the patient. The most common was to attend different patients and procedures using the same gloves, consistent with other studies on the misuse of gloves.^15^, ^25^

Although delineation between patient zones to address recontamination was studied in Vietnam,^26^ to our knowledge, ours is the first study that sought to quantify the contribution of avoiding recontamination to HH compliance. Our findings are supported by studies in the United Kingdom and Australia where health care workers were observed to touch privacy curtains between HH or glove donning and patient care.^15^, ^27^ In a study based in Ghana, Cronin et al. describe qualitatively how birth attendants' gloved hands were observed touching the patient bed before the delivery.^28^ Loftus et al^29^ demonstrated microbiological recontamination of hands at the point of care despite high levels of self-reported HH compliance, indicating the relevance of recontamination in infection transmission. Recontamination may be an indication that there is a lack of understanding of the definition of the WHO 5MHH in its attempt to direct an approach to HH action at times when recontamination risk within or between patients has been established. Future versions of the WHO HH Observation Form could add a recontamination option for the “missed” HH opportunities (when compliance was not met), which would allow for recontamination to be monitored for both implementation and research purposes.

The contribution of avoiding recontamination to overall HH compliance in our study calls for further research, to investigate its importance in other contexts, its drivers, and its direct contribution to HAIs.^7^ Acknowledging the avoidance of recontamination as a distinct behavior and incorporating its measurement into existing tools for observing compliance, such as the WHO HH audit tool, would help quantify this problem and inform interventions to tackle it.

Our analyses revealed that variation in behavior was much larger within individuals than between individuals, suggesting that varying factors, such as availability of materials and workload, may be more important drivers than individual psychological determinants and that behavior-change strategies need to be tailored to actual practices and contexts.^30^, ^31^ It is important to note that these findings were generated in settings with limited resources; hence, in settings with more stable resources, practices may be more habitual. Future studies could further investigate this.

We monitored health care workers’ behavior using state-of-the-art time-and-motion methods that have rarely been employed in low-resource settings.^32^ This allowed us to investigate compliance with the complete sequence prescribed by the WHO guidelines on HH as well as each individual behavior and behavior sequence. It also reduced the risk of observer bias, because HH opportunities were identified retrospectively in a standardized way rather than relying on observer judgment.

Our study had some potential limitations. A residual Hawthorne effect may have caused overestimation of compliance, despite blinding attendants to the study purpose in all but 1 facility. The 13% of opportunities with incomplete hand hygiene or glove information might not be random, as they may have occurred when procedures were rushed and HH more difficult, leading us to overestimate compliance.^33^ In 5 of 336 observation sessions, we did not have data on attendance of new patients and assumed that the same woman was attended throughout, potentially underestimating opportunities for HH and overestimating compliance.

In conclusion, in this time-and-motion study of hand hygiene and glove practices in the 10 highest-volume labor wards in Zanzibar, we found, as did previous studies, low compliance with WHO HH guidelines. The major addition of this study is that it revealed the potential effect of recontamination, after initial washing/rubbing and donning gloves, on infection risk and the importance of including this as a separate item in HH measures. Additionally, variability in this behavior seems to reside primarily within individuals across opportunities. Reducing the threat of HAIs in mothers and newborns calls for further research into drivers of recontamination and effective behavior-change strategies to tackle it.

## Acknowledgments

We thank the Ministry of Health of Zanzibar for their participation and engagement in this study. A special thanks to Rukaiya M. Said, Mwanafatima Ali Mohammed, Bijuma Mkubwa Abdallah, and Asya Hati Vuai who collected all the data. We also thank Marina Daniele for participating in the consultation exercise aimed at refining the definition of opportunity. Finally, we thank Daniel Powell and David Macleod for their support in data management.

## Appendices

All appendices are available from https://doi.org/10.17037/DATA.00000778.

### Appendix A

Facilities description.

### Appendix B

Actions that indicated a new hand hygiene opportunity and were outside of the workflow.

### Appendix C

Numerator and denominator definitions for each outcome combination reported in the Methods section.

### Appendix D

Hand hygiene compliance by observer and shift.

### Appendix E

Duration and technique of hand rubbing/washing.

### Appendix F

Sequence of actions preceding the first aseptic procedure in the delivery flow.

### Appendix G

Patterns of glove behavior under Sequence 9 (from Fig 2).
